# Supplementary material for: Mechanical Signaling Regulates DNA Methylation to Maintain Muscle Stem Cell Quiescence
Source: bioRxiv. 2026 May 17:2026.05.15.725521. Preprint. [Version 1] doi: 10.64898/2026.05.15.725521 (PMC13192721; doi:10.64898/2026.05.15.725521)
Supplement: Supplement 1 [file NIHPP2026.05.15.725521v1-supplement-1.pdf]

# Supplementary Figure 1. Inhibition of RhoA *in vitro* accelerates MuSC activation

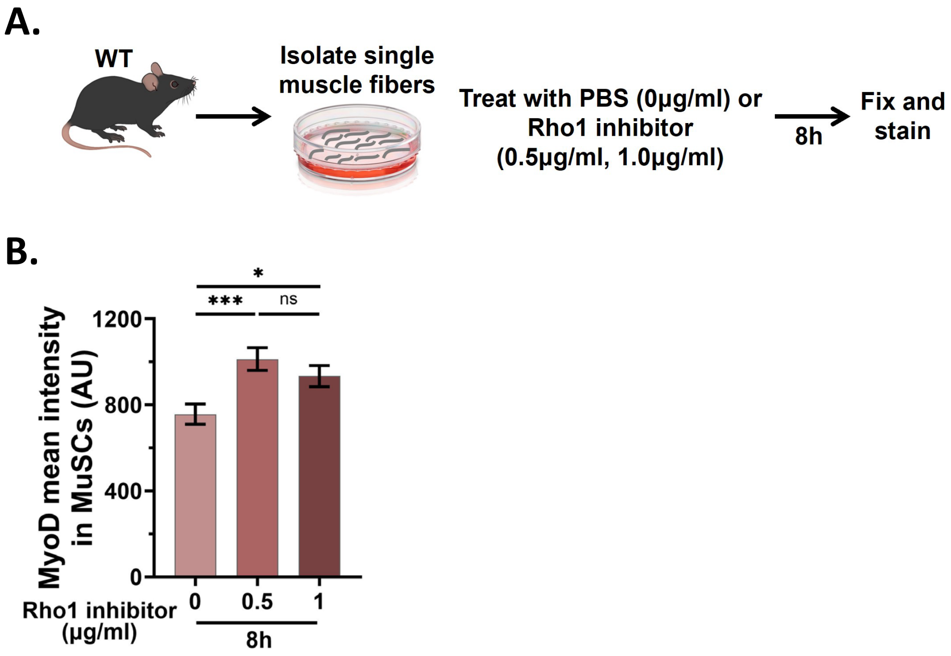


**Supplementary Figure 1. Inhibition of RhoA *in vitro* accelerates MuSC activation**
**Related to Figure 2.**
(A) Schematic of the experimental design.
(B) Quantification of MyoD intensity in MuSCs on isolated WT myofibers that were treated immediately after isolation with increasing concentrations of a RhoA inhibitor (Rho1) for 8 hours (n = 4).

# Supplementary Figure 2. RhoA-dependent transcriptional and epigenetic programs define MuSC state

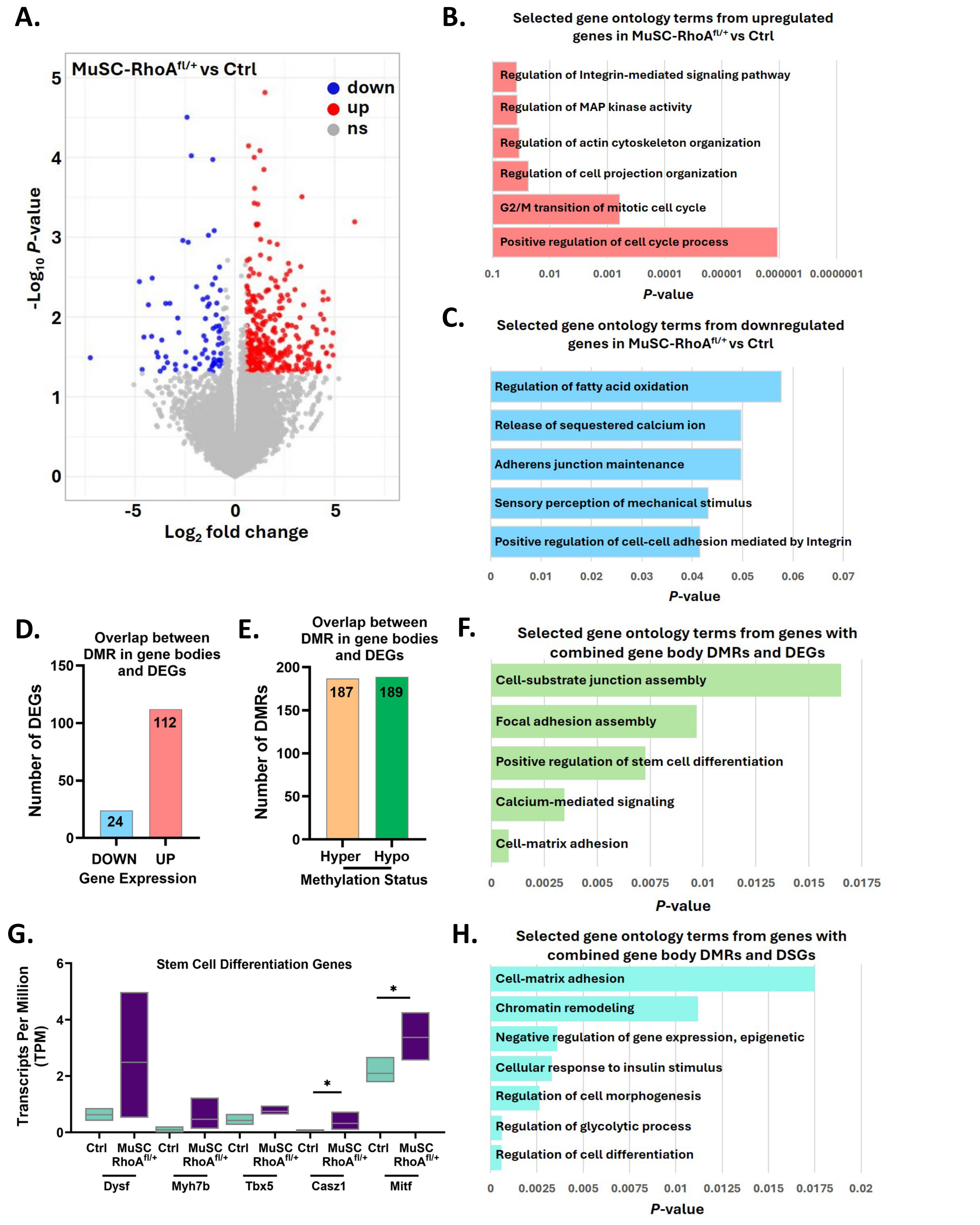

**Supplemental Figure 2. RhoA-dependent transcriptional and epigenetic programs define MuSC state Related to Figure 3.**

(A) Volcano plot of RNA-seq data showing upregulated and downregulated genes in freshly isolated MuSCs from MuSC-RhoA$^{fl/+}$ muscle compared to control.

(B and C) Gene Ontology (GO) analysis of upregulated (B) and downregulated (C) genes.

(D) Distribution of DEGs overlapping with gene body DMRs, categorized as upregulated or downregulated.

(E) Distribution of gene body DMRs overlapping with DEGs, categorized as hypermethylated or hypomethylated.

(F) GO analysis of genes containing both gene body DMRs and differential expression.

(G) Representative stem cell differentiation genes containing both gene body DMRs and differential expression.

(H) GO analysis of genes containing both gene body DMRs and DSGs.

# Supplementary Figure 3. RhoA-dependent mechanical signaling regulates Dnmt3A expression in MuSCs

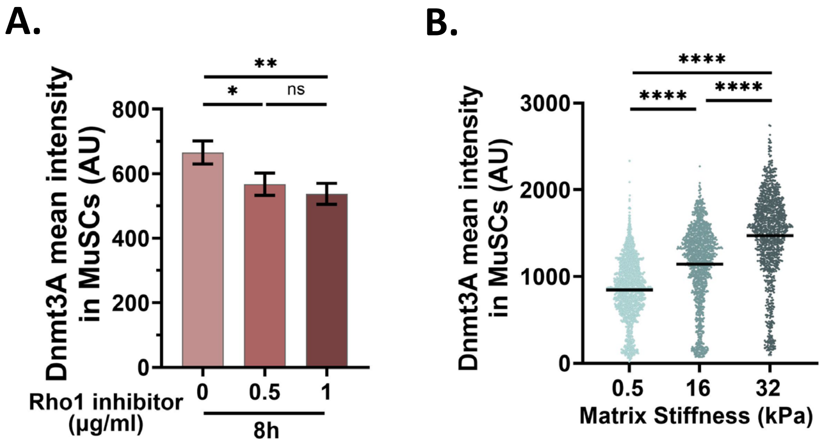


**Supplementary Figure 3. RhoA-dependent mechanical signaling regulates Dnmt3A expression in MuSCs**

**Related to Figure 4.**

(A) Quantification of Dnmt3A intensity in WT MuSCs treated immediately after isolation with increasing concentrations of a RhoA inhibitor for 8 hours (n = 4).

(B) Quantification of Dnmt3A intensity in SCs plated on matrices of defined stiffness (0.5, 16 and 32 kPa) for 10 hours (n=4).

Each dot in bar graphs represents an individual mouse; each dot in dot plots represents an individual MuSC, with the central line indicating the median. Statistical significance was determined using an unpaired two-tailed Student's t test. Error bars, mean $\pm$ SD; *p<0.05, **p < 0.01, ****p < 0.0001, ns-not significant.

# Supplementary Figure 4. Dnmt3A loss phenocopies RhoA depletion in MuSCs

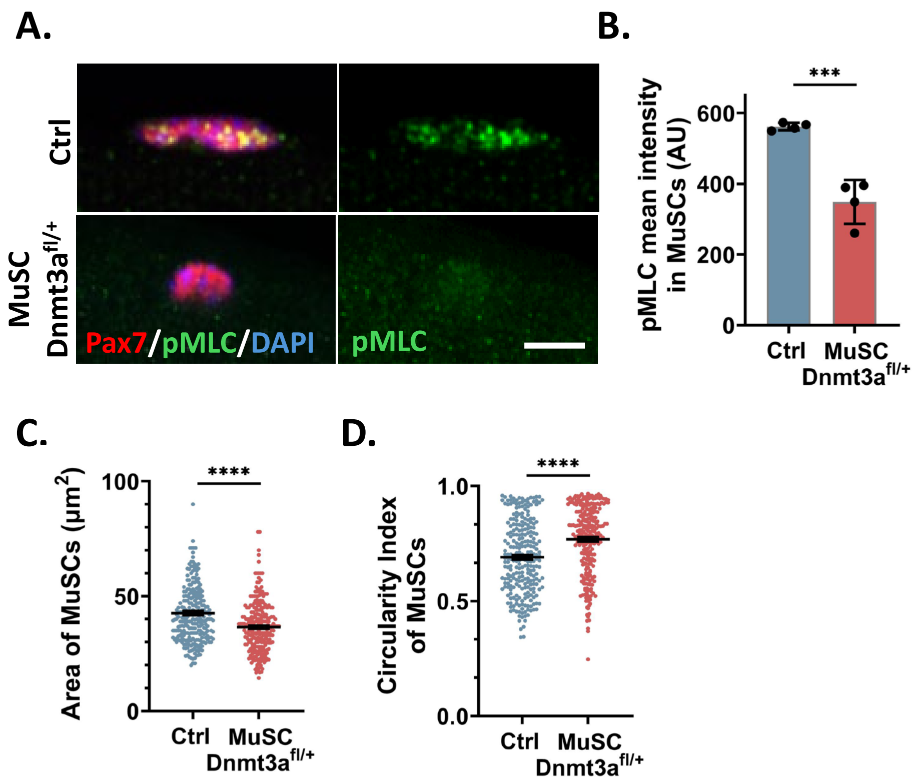


**Supplementary Figure 4. Dnmt3A loss phenocopies RhoA depletion in MuSCs**
**Related to Figure 5**
(A and B) Representative images (A) and quantification (B) of pMLC intensity in MuSCs on control and MuSC-Dnmt3A$^{fl/+}$ myofibers (n = 4).
(C and D) Quantification of cell area (C) and circularity (D) of MuSCs on control and MuSC- Dnmt3a$^{fl/+}$ myofibers.
Each dot in bar graphs represents an individual mouse; each dot in dot plots represents an individual MuSC. Statistical significance was determined using an unpaired two-tailed Student's t test. For the bar graph, Error bars, mean $\pm$ SD; For the dot plots, Error bars, mean $\pm$ SEM; ***p < 0.001, ****p < 0.0001; scale bars, 5μm in (A).

# Supplementary Figure 5. SP1 inhibition *in vitro* promotes MuSC activation and reduces Dnmt3A expression

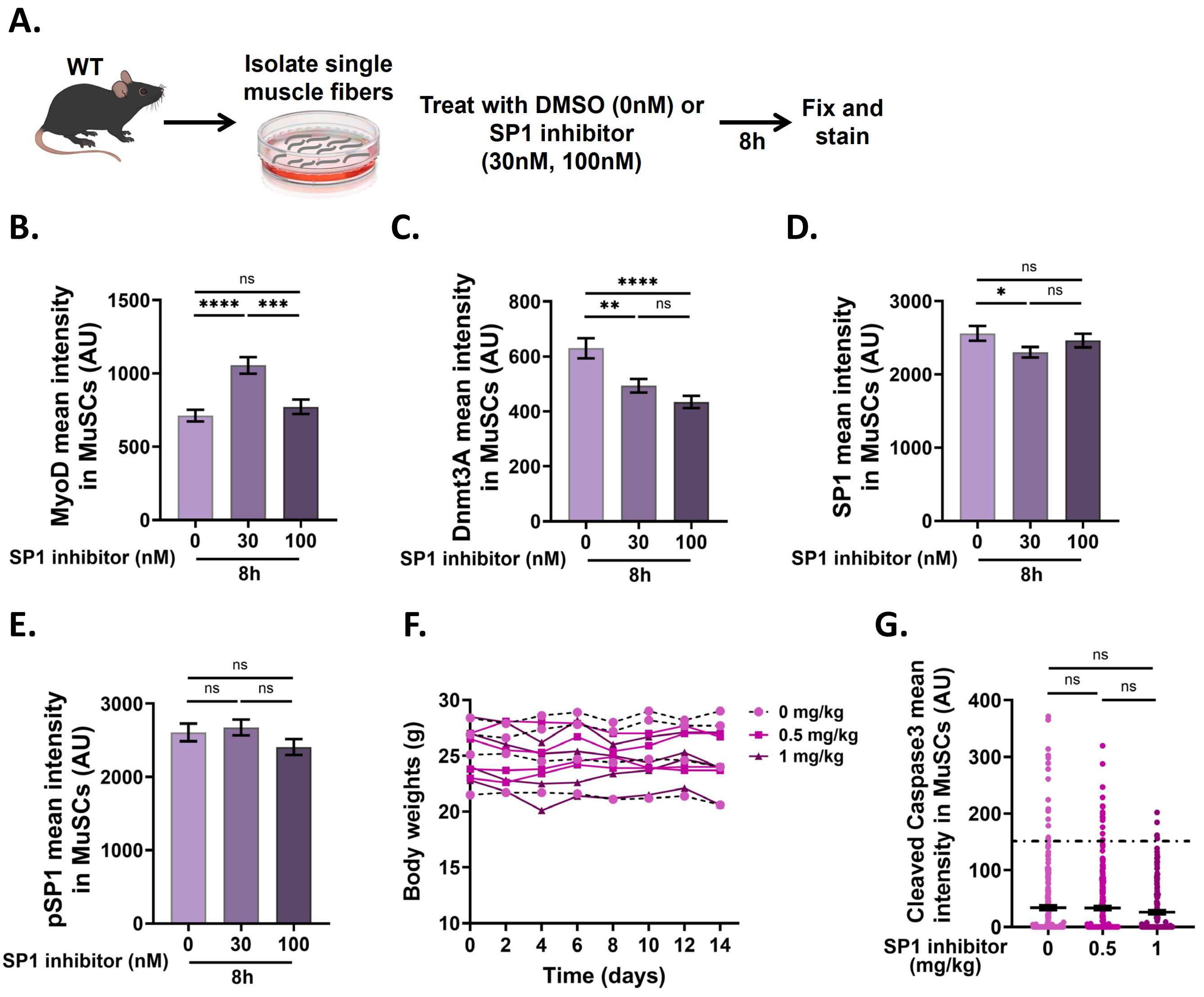


**Supplementary Figure 5. SP1 Inhibition *in vitro* promotes MuSC activation and reduces Dnmt3A expression**

**Related to Figure 7.**

(A) Schematic of the experimental design.

(B - E) Quantification of MyoD intensity (B), Dnmt3A intensity (C), SP1 intensity (D) and pSP1 intensity in WT MuSCs on isolated myofibers treated with vehicle or SP1 inhibitor *in vitro* for 8 hours (n = 3).

(F) Body weight of mice treated with indicated doses (0, 0.5 and 1mg/kg) of SP1 inhibitor for 14 consecutive days. Each line represents an individual mouse measured every other day (n = 3).

(G) Quantification of cleaved caspase 3 intensity in MuSCs on isolated myofibers from vehicle and SP1 inhibitor treated mice. Each dot in the dot plot represents an individual MuSC, with the error bar, mean ± SEM (n = 3). Statistical significance was determined using an unpaired two-tailed Student's t test. For the bar graphs, Error bars, mean ± SD; *p<0.05, **p < 0.01, ***p < 0.001, ****p < 0.0001, ns- non significant.
